# Characterization of the aroma profile of fermented chickpea (*Cicer arietinum* L.) aquafaba by GC-MS and sensory analysis

**DOI:** 10.1016/j.crfs.2026.101333

**Published:** 2026-02-02

**Authors:** Lea Mehren, Rabea Hondrich, Fabian Maretzky, Matthias Wüst, Andreas Schieber, Nadine Schulze-Kaysers

**Affiliations:** a Institute of Nutritional and Food Sciences, Molecular Food Technology, Faculty of Agricultural, Nutritional and Engineering Sciences, University of Bonn, Bonn, Germany; b Institute of Nutritional and Food Sciences, Food Chemistry, Faculty of Agricultural, Nutritional and Engineering Sciences, University of Bonn, Bonn, Germany

**Keywords:** Chickpea aquafaba, Aroma, Fermentation, GC-MS, CATA

## Abstract

The utilization of aquafaba, the cooking water of chickpeas, as a vegan egg-replacer is limited by its beany aroma. To improve its sensory properties, aquafaba was subjected to fermentation with the basidiomycetes *Antrodia xantha* and *Lentinula edodes,* and its aroma was examined using gas chromatography-mass spectrometry and check-all-that-apply analysis by a trained sensory panel. The study revealed that the mushrooms synthesized distinctive aroma compounds, with linalool and 1-undecanol being detected exclusively during the fermentation with *A. xantha* and 1-octanol, carveol and 2,4-nonadienal being detected exclusively during the fermentation with *L. edodes*. The latter two substances were not detected in the pre-culture, suggesting that their synthesis occurs during the fermentation of aquafaba with *L. edodes*. The initial characteristic beany odor impression was reduced by fermentation, the aroma profile was significantly altered during the fermentation process with *L. edodes*. While the smell of aquafaba was predominant at the beginning of fermentation, it changed over time to a sweet, roasty aroma, and finally to a woody aroma in the last third of fermentation. The time-dependent changes in aroma compounds and sensory attributes suggest that varying the fermentation duration may enable specific applications.

## Highlights

• First characterization of aroma profile in side stream fermented with *A. xantha.*
• Analytical and sensory data clearly distinguished fermented aquafaba samples.
• During *L. edodes* fermentation, aroma shifted from aquafaba to sweet to woody.
• The product typical beany aroma decreased during fermentation.

## Graphical abstract

**Figure:**
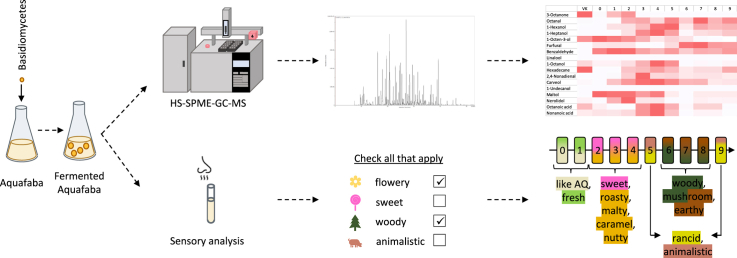

## 1 Introduction

The growing demand for high-quality plant-based proteins has resulted in an increase in the cultivation and consumption of pulses (OECD-FAO Agricultural Outlook, 2025, 2025). These alternative sources offer high nutritional value while reducing environmental impact, as their cultivation is associated with lower greenhouse gas emissions, water consumption, and artificial fertilizer use (OECD-FAO Agricultural Outlook, 2025, 2025; Drewnowski and Conrad, 2024; Gustafson, 2017; Stagnari et al., 2017). However, within the context of sustainable food production, it is essential to investigate the potential of utilizing by-products of pulse production, such as aquafaba. Aquafaba, derived from the Latin words “aqua” for water and “faba” for bean, refers to the cooking water of various pulses, including chickpeas, lentils, peas, and various beans (He et al., 2021). It has been shown to possess functional properties, including foaming, emulsifying, and gelling properties (He et al., 2021; Mustafa et al., 2019; Buhl et al., 2019; Yazici et al., 2022). These properties and the composition of aquafaba vary depending on the source and type of legume (Sahin et al., 2025). Aquafaba from chickpeas has most widely been studied. Processing 1 kg of chickpeas yields approximately 0.6–1 kg of aquafaba (Mustafa et al., 2019). Differences in composition and properties have also been observed between different cultivars of chickpea (He et al., 2019).

Literature about the aroma composition of aquafaba is scarce. The presence of aroma compounds in aquafaba is dependent on the source of legumes utilized in the production process, thereby influencing its composition. This group comprises many chemical classes, including aldehydes, alcohols, ketones, and terpenes, among others (Karolkowski et al., 2021). Nevertheless, aquafaba generally exhibits promising functional properties, making it a suitable replacement for egg-white or milk as a low-allergy ingredient. It has therefore attracted attention, especially within the vegan and gluten-free communities, where it has gained popularity in plant-based recipes (Mustafa et al., 2019). Various vegan sweet dishes, including meringues (Tufaro and Cappa, 2023), chocolate mousse (Mehren et al., 2025), ice cream (Erem et al., 2024) and sponge cake (Mustafa et al., 2018) are now being produced using aquafaba.

However, the application of aquafaba in food is limited by its beany-aroma, which may result in lower consumer acceptance, particularly in sweet dishes (Noordraven et al., 2021). Fermentation offers a potential solution, as microorganisms are responsible for synthesizing flavor compounds (Fritsche, 2016). The composition of these aroma-active substances is influenced by numerous factors, including substrates, microorganisms, and duration of the fermentation process (Chisti et al., 2010). Basidiomycetes are known to produce flavor compounds either de novo or through biotransformation (Lomascolo et al., 1999). Specifically, the mushroom *Lentinula edodes*, commonly referred to as the shiitake mushroom, has been the subject of prior research investigating the extraction of aromatic substances from by-products (Baktemur et al., 2020; Zhang et al., 2014). During these fermentations, a reduction in the intensity of the substrates’ characteristic aromas was observed, accompanied by the emergence of new aromatic compounds (Zhang et al., 2015). This has also been demonstrated in our previous study where the fermentation of aquafaba with two basidiomycetes, *Antrodia xantha* and *Lentinula edodes,* resulted in a reduced perception of the characteristic beany off-flavor and the appearance of new olfactory impressions, as described by a trained sensory panel (Mehren et al., 2025).

In continuation of this work, the present study aimed to determine the composition of the aromatic compounds formed during aquafaba fermentation with the basidiomycetes *Antrodia xantha* and *Lentinula edodes*. Changes in volatile composition during fermentation were monitored over nine days using headspace solid-phase microextraction gas chromatography mass spectrometry (HS-SPME-GC-MS). With regard to the application in food, the olfactory impression of the samples was characterized by a trained sensory panel using the check-all-that-apply (CATA) rapid method. The combined analysis of analytical and sensory data allowed conclusions about the contribution of individual aroma compounds to the perceived aroma of fermented aquafaba and its potential for the application in food products.

## 2 Materials and methods

### 2.1 Materials

Hexyl alcohol (98%) and 3-hydroxy-2-methyl-4-pyrone (99%) were purchased from Thermo Fisher Scientific (Waltham, Massachusetts, USA). Linalool (±) (97%), α-terpineol (±) (96%), nerolidol *E + Z* (≥97%), nonanal (97%), and 1-octanol (99%) were obtained from Alfa Aesar, Thermo Fisher GmbH (Kandel, Germany). Tetrahydrofurfuryl alcohol (99%), *(E, E)*-2,4-nonadienal (≥89%), 1-undecanol (99%), hexadecane (99%), benzaldehyde (≥99%), 3-octanone (≥98%), furfural (99%), octanal (99%), and (−)-carveol (97%) were purchased from Sigma Aldrich (St. Louis, Missouri, USA). Octanoic acid (≥99%), 1-heptanol (99%), 1-octen-3-ol (±) (>97%) and agar-agar were supplied from Merck GmbH (Darmstadt, Germany). Glacial acetic acid (99.9%), acetonitrile (≥99,9%), water (HPLC LC-MS grade) and methanol (≥99,9%) were obtained from VWR International (Radnor, Pennsylvania, USA). Nonanoic acid (≥99.5%) was sourced from Fluka Chemie GmbH (Buchs, Switzerland) and sodium chloride (99%) was purchased from Th. Geyer GmbH & Co. KG (Renningen, Germany). Filbertone was supplied from Dreidoppel GmbH (Langenfeld, Germany). The malt extract was obtained from Carl Roth GmbH & Co. KG (Karlsruhe, Germany).

Chickpeas, variety *Azkan* (Turkey) were purchased from Bode Naturkost, Horst Bode Import-Export GmbH (Hamburg, Germany). Basidiomycetes strains *Antrodia xantha* (AXA) and *Lentinula edodes* (Berk.) Pegler (LED) (strain number 389.89) were kindly provided by the Institute of Food Chemistry and Food Biotechnology (Justus-Liebig-University, Gieβen, Germany).

### 2.2 Sample preparation

**Fig. 1:**
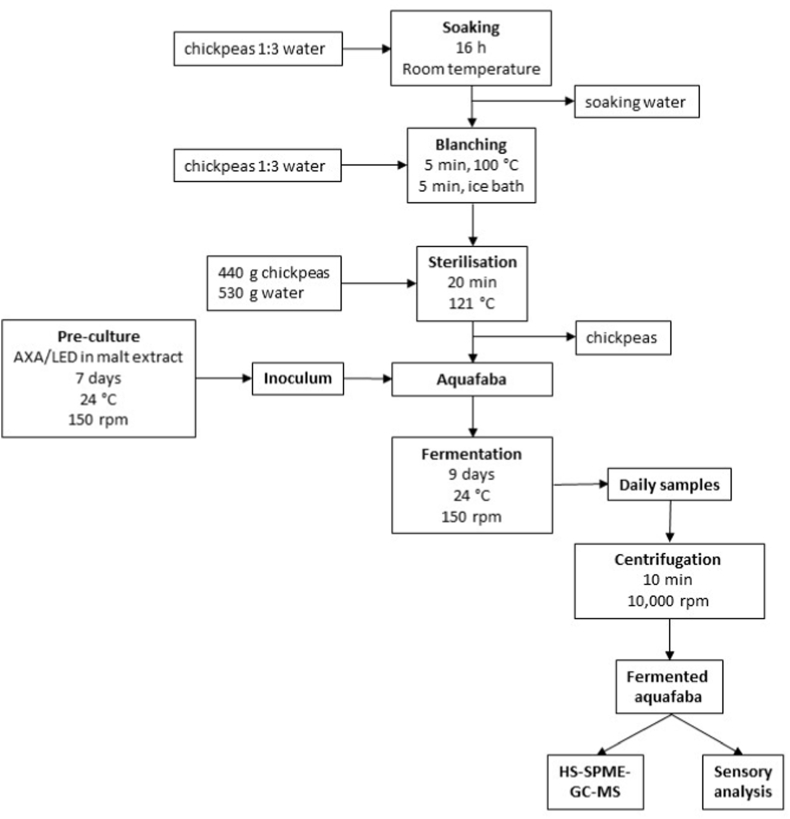
Production process of fermented aquafaba for GC-MS and sensory analysis.

The production of aquafaba was conducted according to a previously standardized procedure. To this end, chickpeas were soaked in water at a ratio of 1:3 for 16 h at ambient temperature. After removal of the soaking water, the chickpeas were cooked in a Thermomix TM6 (Vorwerk Deutschland Stiftung & Co. KG, Wuppertal, Germany) at 100 °C for 5 min, with the addition of fresh water at a ratio of 1:3. The cooking water was then discarded and the chickpeas were subsequently cooled in an ice bath for 5 min. Afterwards, 440 g of chickpeas and 530 g of water were transferred into 1 L bottles and sterilized at 121 °C for 20 min in an autoclave. The bottles were cooled overnight and the aquafaba was separated from the chickpeas. Fig. 1 shows the production process, fermentation and subsequent analyses.

### 2.3 Fermentation of aquafaba

The fermentation of aquafaba was performed as outlined by Mehren et al. (2025). In brief, the mushrooms were cultivated on a malt extract agar plate (20 g/L malt extract, 15 g/L agar) at 24 °C in an incubator. Once 80% of the plate has been covered with the mycelia, a piece of the covered malt extract was transferred into 100 mL of a 2% (*w/v*) malt extract pre-culture (after a period of seven days for AXA and eleven days for LED). The pre-culture was incubated at 24 °C and 150 rpm for a period of seven days. For the subsequent submerged fermentation 10 mL of homogenized inoculum was added to 98 mL of aquafaba and 2 mL sugar solution (1 mg/mL). The aquafaba was fermented for 9 days at 24 °C and 150 rpm and samples were taken daily starting with the inoculation. The samples were centrifuged for 10 min at 10000 rpm to separate the fermented aquafaba from the mushroom. The supernatant was then stored at −20 °C until further analyses.

### 2.4 Headspace solid-phase microextraction gas chromatography-mass spectrometry

The determination of aroma-active compounds in the fermented samples was conducted by headspace solid-phase microextraction gas chromatography-mass spectrometry (HS-SPME-GC-MS). For this purpose, 2 mL of aquafaba and 1 mL of a 20% (*w/v*) sodium chloride solution were transferred into 10 mL headspace vials. HS-SPME was carried out using a divinylbenzol/carboxen/polyethylsiloxan fiber (DVB/CAR/PDMS, 50/30 μm, 2 cm) (Supelco, Sigma Aldrich, St. Louis, Missouri, USA). The samples were incubated at 80 °C for 5 min with agitation (250 rpm, 2 s on time, 4 s off time), followed by the extraction at 80 °C for 30 min. Subsequently, the extracted volatiles were desorbed in the injection port at 250 °C for 5 min and injected in splitless mode. The chromatographic separation was performed using an Agilent 7890B GC (Agilent Technologies, Palo Alto, California, USA) with an Optima WAXplus® (30 m × 250 μm) (Machery-Nagel, Düren, Germany) capillary column with helium as the carrier gas at a constant flow of 1.5 mL/min. The initial oven temperature was 40 °C and held for 6 min. The temperature was increased to 250 °C at 5 °C/min and maintained for 5 min. MS detection was performed using a time-of-flight mass spectrometer (Markes International Ltd., Bridgend, UK) operating in electron ionization mode at −70 eV. The mass spectrum ranged from 35 *m/z* to 300 *m/z*. The temperature of the transfer line and the ion source was set at 250 °C. Data analysis was conducted using TOF-DS (Markes International Ltd., Llantrisant, RCT, UK). The identification of volatiles was achieved by comparison of their retention times and peak areas with those of standard substances and the NIST Mass Spectral Library (National Institute of Standards and Technology, Gaithersburg, Maryland, USA). Quantification was carried out by external calibration using standard substances. Measurements were carried out in triplicate.

### 2.5 Sensory evaluation

#### 2.5.1 Panel training

The sensory analysis was carried out using a check-all-that-apply (CATA) survey. In total, 17 panelists were trained in 7 sessions (mean age = 35; range: 21–63; 17 female). The training comprised smell identification and recognition tests. The reference substances used during the trainings are listed in Table 1. Each panelist was provided with a set of five olfactory samples containing solutions of different aroma substances, namely 1-hexanol, 1-octen-3-ol, linalool, maltol, and α-terpineol. The panelists were instructed to smell the samples twice a day, thereby committing the olfactory impression to their sensory memory. After one week, recognition abilities were assessed through the implementation of a modified memory game. It was based on a matching test (DIN EN ISO 8586), wherein the panelists were also asked to name the perceived aroma. Further training was provided in the form of a triangle test (DIN EN ISO 4120), in which the aromas were presented at two distinct concentration levels. A product specific training was finally conducted, in which aquafaba samples spiked with the aforementioned aroma compounds were served to the panelists. These samples were evaluated with the final questionnaire.

**Table 1:** Sensory attributes of the CATA questionnaire and the appropriate reference substances.

Attributes	References
aquafaba-like	aquafaba
mushroom-like	1-octen-3-ol (1% (*w/v*))
malty	malt beer without alcohol
sweet odor	maltol (1% (*w/v*))
nutty	filbertone (1% (*w/v*))
earthy	wet forest soil
woody	α-terpineol (1% (*w/v*))
grass	1-hexanol (1% (*w/v*))
fresh	1-hexanol (1% (*w/v*))
flowery	linalool (1% (*w/v*))
musty	α-terpineol (1% (*w/v*))
roasty	caramel from sugar
caramel	caramel from sugar
rancid	filbertone (1% (*w/v*))
spicy	no reference
buttery	no reference
animalistic	no reference

#### 2.5.2 Sensory evaluation

The evaluation of the fermented samples was conducted in the sensory laboratory at the Institute of Nutritional and Food Sciences of the University of Bonn in five sessions (n = 17). In four of these sessions, four fermented aquafaba samples were evaluated, while in one session, four fermented samples and the unfermented aquafaba were studied. The fermented aquafaba samples were thawed at ambient temperature on the day of evaluation. Aliquots of 20 mL were transferred into brown glass containers and sealed with a screw cap, thereby enabling the accumulation of aroma compounds within the headspace. The panelists received the samples in a randomized order, with each sample being coded with a three-digit number. The evaluation was conducted using a check-all-that-apply (CATA) questionnaire. The attributes were listed in a randomized order for each sample and are displayed in Table 1. The panelists were requested to neutralize their sense of smell by smelling either coffee beans or their own skin after each sample. For this purpose, an automatic timer with a duration of 30 s was implemented. The data was collected using the sensory software RedJade® (Sensory Solutions, LLC, Pleasant Hill, California, USA).

### 2.6 Statistical analyses

Statistical analyses were performed using Microsoft Excel, version 2016 and XLStat Sensory, version 2019.2.1 (Lumivero, Denver, Colorado, USA). To investigate significant differences between the levels of aroma compounds during the fermentation process, a one-way analysis of variance (ANOVA) followed by Tukey's honest significance test (p < 0.05) was performed. The check-all-that-apply data set was analyzed using the Cochran's Q-Test (p < 0.05) and a correspondence analysis. A partial least squares discriminant analysis was conducted to ascertain whether the two mushrooms can be distinguished by the combination of the analytical and sensory data. To identify relations between aroma compounds and sensory attributes, the data was standardized using z-transformation and a principal component analysis was conducted.

## 3 Results and discussion

### 3.1 Characterization of selected aroma compounds in aquafaba and fermented aquafaba

Table 2, Table 3 show the results of the aroma analysis for 20 volatiles that were identified. Twelve of the 20 volatiles analyzed were detected in unfermented aquafaba. Four of them – 1-hexanol, 1-octanol, nonanal, and benzaldehyde – had already been identified in chickpea aquafaba (Noh and Lee, 2024). Since aquafaba is the cooking water of chickpeas, its aroma compounds either derive from the chickpeas or are produced during thermal processing. While 1-heptanol and benzaldehyde were detected in raw soaked chickpeas, furfural and maltol were detected only in sterilized chickpeas (Noordraven et al., 2021). These two aromatic substances may have been produced as a consequence of the Maillard reaction during the sterilization process of chickpeas (Lebensmittelchemie and Springer Berlin Heidelberg, 2008; Noordraven et al., 2021). Acetic acid, octanoic acid (Xu et al., 2019) and hexadecane (Rembold et al., 1989) were also previously detected in chickpeas.

**Table 2:** Concentrations of the aroma compounds (in μg/L) for aquafaba fermented with *Antrodia xantha* and the unfermented aquafaba during the fermentation period measured by HS-SPME-GC-MS.

Aroma compound	AXAVK	AXA0	AXA1	AXA2	AXA3	AXA4	AXA5	AXA6	AXA7	AXA8	AXA9	Aquafaba
3-Octanone	20.1	8.9	19.5	18.2	10.9	8.9	8.0	n.d.	n.d.	15.1	3.2	n.d.
Octanal	12.4	5.4	5.1	2.9	4.6	2.4	2.0	1.7	n.d.	3.2	2.8	n.d.
1-Hexanol	0.6	n.q.	3.7	3.3	4.3	2.3	0.7	2.0	1.0	n.d.	4.7	3.7
Benzaldehyde	10.4	41.8	31.1	28.3	37.7	35.6	40.7	38.2	41.8	26.9	39.6	24.2
1-Octanol	n.d.	n.d.	n.d.	n.d.	n.d.	n.d.	n.d.	n.d.	n.d.	n.d.	n.d.	n.q.
Hexadecane	n.q.	n.d.	n.d.	n.q.	n.q.	n.q.	n.q.	n.q.	n.q.	n.q.	n.q.	n.q.
2,4-Nonadienal	n.d.	n.d.	n.d.	n.d.	n.d.	n.d.	n.d.	n.d.	n.d.	n.d.	n.d.	n.d.
Carveol∗	n.d.	n.d.	n.d.	n.d.	n.d.	n.d.	n.d.	n.d.	n.d.	n.d.	n.d.	n.d.
1-Undecanol	3.1	n.q.	0.6	0.9	0.5	0.9	1.0	n.q.	1.6	n.q.	2.3	n.d.
Nerolidol∗	5.1	1.9	18.3	17.0	17.5	16.1	22.4	20.0	36.9	18.5	20.7	n.d.
1-Heptanol	n.d.	n.d.	n.d.	n.d.	n.d.	0.4	n.d.	n.d.	n.d.	n.d.	n.d.	n.q.
1-Octen-3-ol∗	10.7	8.9	9.3	4.8	8.6	2.7	1.3	2.5	n.q.	3.4	n.q.	n.d.
Furfural	n.d.	302.2	30.4	54.7	54.9	205.2	620.6	520.3	8.8	65.5	201.0	106.4
Linalool∗	41.0	n.d.	40.7	39.4	24.5	14.7	97.5	88.1	n.q.	12.7	n.q.	n.d.
Maltol	n.d.	3003.4	2935.1	2273.4	2528.0	2718.6	3429.8	3403.3	3346.5	3097.7	3354.0	3578.1
Octanoic acid	3687.5	3084.5	3381.9	1610.0	1594.6	5181.2	2356.5	2284.0	2072.0	1907.7	1559.0	1835.9
Nonanoic acid	6141.7	4546.7	4821.4	1730.4	2167.9	7014.7	2717.4	2402.8	2553.3	1275.4	1149.0	1639.1

Nonanal, acetic acid and α-terpineol were identified in all samples but not quantified; n.d. = not detected; n.q. = not quantified; ∗ the enantiomeric ratio was not determined.

**Table 3:** Concentrations of the aroma compounds (in μg/L) for aquafaba fermented with *Lentinula edodes* and the unfermented aquafaba during the fermentation period measured by HS-SPME-GC-MS.

Aroma compound	LEDVK	LED0	LED1	LED2	LED3	LED4	LED5	LED6	LED7	LED8	LED9	Aquafaba
3-Octanone	22.5	3.9	10.6	16.1	n.d.	n.d.	n.d.	n.d.	n.d.	n.d.	n.d.	n.d.
Octanal	2.6	2.3	2.4	8.0	8.6	5.1	11.1	5.9	13.1	7.6	10.5	n.d.
1-Hexanol	0.9	0.8	4.0	4.0	8.1	16.9	18.0	3.2	10.7	8.5	9.4	3.7
Benzaldehyde	n.d.	39.4	47.7	46.4	28.6	21.8	31.7	15.0	25.5	24.0	31.4	24.2
1-Octanol	5.1	2.6	3.9	4.8	7.0	10.5	8.7	0.6	1.6	1.2	2.1	n.q.
Hexadecane	n.q.	n.d.	n.d.	n.q.	n.q.	n.q.	n.q.	n.d.	n.q.	1.1	n.q.	n.q.
2,4-Nonadienal	n.d.	n.d.	2.3	10.1	28.7	13.4	14.8	7.6	6.3	6.4	5.1	n.d.
Carveol∗	n.q.	n.d.	4.2	7.4	10.5	10.8	8.5	5.1	5.4	1.5	1.8	n.d.
1-Undecanol	n.d.	n.d.	n.d.	n.d.	n.d.	n.d.	n.d.	n.d.	n.d.	n.d.	n.d.	n.d.
Nerolidol∗	n.q.	n.d.	1.5	2.2	0.3	n.q.	n.q.	n.d.	n.d.	n.d.	n.d.	n.d.
1-Heptanol	n.d.	n.d.	n.d.	1.0	3.2	4.1	1.6	n.q.	0.8	1.0	1.5	n.q.
1-Octen-3-ol∗	8.3	11.4	10.2	8.5	6.0	2.0	3.5	2.4	1.0	0.4	n.d.	n.d.
Furfural	10.6	105.8	116.7	116.9	14.8	10.8	150.9	592.4	637.2	428.7	412.5	106.4
Linalool∗	n.d.	n.d.	n.d.	n.d.	n.d.	n.d.	n.d.	n.d.	n.d.	n.d.	n.d.	n.d.
Maltol	n.d.	3302.8	3122.2	3127.9	2241.3	2889.2	531.4	531.5	551.9	543.0	547.0	3578.1
Octanoic acid	3446.5	2349.5	2070.6	2108.3	4245.1	5181.2	3689.3	2195.4	2958.2	2066.7	2072.3	1835.9
Nonanoic acid	3477.0	2485.0	2419.3	2672.0	5359.1	7014.7	3685.4	1530.8	1889.6	1635.2	1593.9	1639.1

Nonanal, acetic acid and α-terpineol were identified in all samples but not quantified; n.d. = not detected; n.q. = not quantified; ∗ the enantiomeric ratio was not determined.

In the pre-culture of the AXA sample, 13 of the 20 analyzed aroma compounds were detected, whereas 15 of the analyzed aroma compounds were found in the LED pre-culture. To the best of our knowledge, there is no research available concerning the aroma of *Antrodia xantha* or its culture broth. Therefore, the data are compared with those of other *Antrodia species*. *Antrodia camphorata* has been identified as a potential source for the extraction of natural aroma compounds (Xia et al., 2011; Lu et al., 2011; Liu et al., 2008). The following aroma compounds were identified in the culture broth of *Antrodia camphorata*: 1-octen-3-ol, 1-hexanol, 3-octanone, nonanal, α-terpineol, linalool, nerolidol, and benzaldehyde (Lu et al., 2011). The analytes 1-octanol (Lu et al., 2011) and furfural (Liu et al., 2008) were also detected in *Antrodia camphorata* but not found in AXA pre-culture. The formation of aromatic substances by basidiomycetes depends on numerous parameters, including substrate composition, time of harvest, and enzymatic activity (Baktemur et al., 2020; Cho et al., 2003). As the enzyme system of AXA has only scarcely been described, it is not possible to draw any conclusions.

For LED, the aroma compounds 3-octanone, octanal, acetic acid, 1-octen-3-ol, furfural, 1-octanol, octanoic acid and nonanoic acid were previously detected in LED culture broth (Li et al., 2019), while 1-hexanol was found in LED fermented wort (Zhang et al., 2014). In addition, nonanal (Hou et al., 2021) and hexadecane (Zhang et al., 2020a) were detected in fresh shiitake mushrooms, and nerolidol was found in dried LED mushrooms (Zhou et al., 2015). Contrary to the results of previous studies demonstrating that LED is capable of producing benzaldehyde, it was not detected in the pre-culture (Li et al., 2019; Zhang et al., 2014).

For both mushrooms, 1-heptanol and maltol were detected throughout the fermentation period but not in the pre-culture. This can be attributed to the addition of aquafaba, in which both aroma compounds were found. Furthermore, it was demonstrated that certain substances are only present in the fermented aquafaba of one of the mushrooms. In this work, it was observed that the production of linalool and 1-undecanol is exclusive to AXA, while 1-octanol, carveol, and 2,4-nonadienal were only detected in LED.

### 3.2 Effects of fermentation on aroma compounds

The concentrations of the analytes exhibited fluctuations during the fermentation process. Maltol, octanoic acid, and nonanoic acid were found in high quantities in all samples of both mushrooms. 1-Octen-3-ol has been identified as a characteristic alcohol in basidiomycetes and has been detected as a major volatile in both LED and *Antrodia species* (Baktemur et al., 2020; Lu et al., 2011; Wu and Wang, 2000), formed via the lipoxygenase pathway through the oxidation of fatty acids (Abraham and Berger, 1994). A significant (p < 0.0001 for both mushrooms) decrease in the concentration of 1-octen-3-ol was observed in the fermented aquafaba for both mushrooms from day 3 to day 9 of the fermentation process. A comparable trend was observed in the solid-phase fermentation of *Antrodia camphorata* (Xia et al., 2011) and during the growth stages of the shiitake mushroom (Cho et al., 2003; Sun et al., 2021). A hypothesis was proposed for the pathway of 1-octen-3-ol in basidiomycetes (Karrer et al., 2021). It was postulated that 1-octen-3-ol can be oxidized to its ketone 1-octen-3-one via alcohol dehydrogenase, which, in turn, can be converted to 3-octanone via ene-reductase and further to 3-octanol by alcohol dehydrogenase. In addition, 3-octanol can be produced directly from 1-octen-3-ol via double bond reductase (Karrer et al., 2021). The aforementioned studies demonstrated a decline in 1-octen-3-ol accompanied by an increase in 3-octanone (Cho et al., 2003; Xia et al., 2011). 3-Octanone was detected in the AXA fermented aquafaba, but its concentrations fluctuated over the fermentation period. Consequently, a direct correlation could not be established. In LED-fermented aquafaba, 3-octanone was detected only during the first two days of fermentation. In the present study, 1-octen-3-one and 3-octanol were not quantified.

Basidiomycetes are known to produce benzaldehyde (Bosse et al., 2013). Although benzaldehyde is already present in unfermented aquafaba, its concentration increased significantly (p < 0.0001 for AXA, and p = 0.036 for LED) at the beginning of fermentation. During the fermentation period, a fluctuation in the concentration was observed, which is comparable to the results of previous studies (Sun et al., 2021). Yet, it remained consistently high and made an important contribution to the volatile composition. Basidiomycetes can produce benzaldehyde from the precursor phenylalanine, which is present in aquafaba (Noh and Lee, 2024), either de novo or by bioconversion (Lomascolo et al., 1999). Another aroma compound that was detected in high concentrations in both fermented samples is furfural. A similar pattern was observed for this volatile and both basidiomycetes. The furfural content initially increased, reaching its peak on fermentation day 5 for AXA and day 7 for LED, before it decreased again.

For the aroma compound maltol differences in content between the two mushrooms became evident during the fermentation process. While the content of maltol in AXA-fermented aquafaba remained consistently high, a significant (p < 0.0001) decrease in maltol content was observed in the LED-fermented aquafaba between days 4 and 5 of fermentation. This may be due to the ability of white rot fungi to cleave aromatic ring systems. This assumption is supported by the structural similarity to catechol, for which the degradation by *Phanerochaete chrysosporium* has been demonstrated (Kato et al., 2024).

The AXA fermented aquafaba is mainly characterized by linalool and nerolidol, which were found in high concentrations over the entire course of fermentation. Furthermore, the odor activity value for linalool was high (OAV>1) during the fermentation, especially from day 1–6, indicating that linalool contributes to the perceived aroma of aquafaba throughout the fermentation process (supplementary data). This is a major difference from the LED sample, where linalool was not detected at all and nerolidol was only found in traces. Fungal terpenoid compounds, including linalool and nerolidol, are formed via the mevalonate pathway (Schmidt-Dannert, 2015). Terpene synthases have been characterized in *Antrodia cinnamomea,* leading to the production of linalool with the substrate GPP (geranyl pyrophosphate) and nerolidol via farnesyl pyrophosphate, which were detected in its mycelium (Lin et al., 2017). However, it is conceivable that these substances are released into the culture medium.

The LED-fermented aquafaba is distinguished from the AXA-fermented sample by the presence of 1-octanol, carveol, and 2,4-nonadienal. Since carveol and 2,4-nonadienal were not detected in both the unfermented aquafaba and the LED preculture, these aroma compounds are likely produced during the fermentation of aquafaba with LED. During the fermentation process, the levels of carveol increased from day 1 to day 4 and then decreased steadily until day 9. Carveol is produced by limonene-6-hydroxylase from limonene and can then undergo further oxidation to carvone (Bouwmeester et al., 1998), which might explain the variations during the fermentation process. This transformation was already observed in basidiomycetes (Sandes et al., 2024). The aroma compound 2,4-nonadienal can be produced enzymatically from linoleic acid via lipoxygenase activity (Tressl et al., 1981; Zhang et al., 2020b). As chickpeas are rich in linoleic acid (Jukanti et al., 2012), it may also be abundant in aquafaba. To the best of our knowledge, direct production of 2,4-nonadienal by basidiomycetes fermentation has not yet been described, but lipoxygenase activity has been detected in *Lentinula edodes* (Sun et al., 2021).

### 3.3 Sensory evaluation of fermented aquafaba

**Fig. 2:**
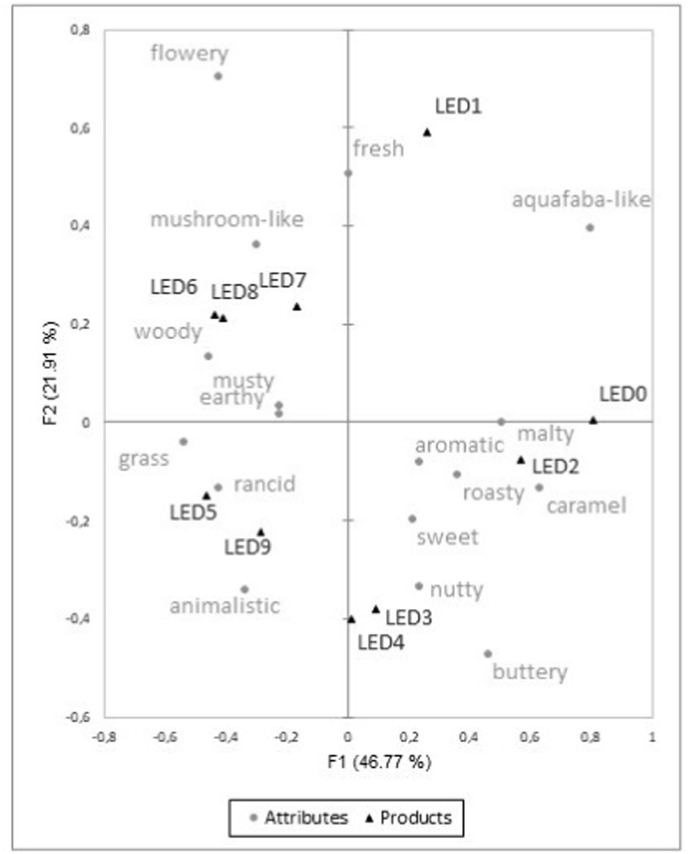
Biplot of the correspondence analysis of the CATA attributes for the LED-fermented aquafaba during fermentation.

In the check-all-that-apply analysis conducted with a trained panel, the frequency of choice of attributes per sample was recorded. The attributes “aquafaba-like” and “mushroom-like” were frequently selected by the panelists to describe the fermented aquafaba samples of both mushrooms. However, there are also differences between aquafaba samples fermented with AXA and those subjected to LED fermentation. In the AXA-fermented aquafaba, the attribute “sweet” was most frequently chosen by the panel to describe the samples throughout the entire fermentation process. Furthermore, the most prevalent attributes identified were “malty”, “nutty”, “caramel” and “roasty”, in terms of frequency. Conversely, for the LED-fermented aquafaba samples, attributes evoking negative connotations, such as “animalistic”, “rancid” and “musty”, were frequently selected. No distinct trend emerged in the frequency of attribute selection during the fermentation process of AXA fermented aquafaba, but for the fermentation with LED. Correspondence analysis of the CATA data for LED-fermented aquafaba, as illustrated in Fig. 2, elucidates this trend. The relationship between attributes and samples can be presented spatially using a contingency table. The presence of points in close proximity to each other serves as an indication of a correlation. It is evident that the samples are separated by the F1 axis based on the fermentation time. The fermentation days 0–4 are located on the right side of the F1 axis, while days 5–9 are positioned on the left. The samples collected on fermentation days 0 and 1 are predominantly characterized by the attribute “aquafaba-like”. It is noteworthy that the frequency of this attribute decreased during the fermentation process. The olfactory profile of the samples from the second and third days of fermentation is characterized by the following attributes: “malty”, “roasty”, “caramel”, “nutty” and “sweet”. The DLG aroma wheel groups these attributes together in an aroma family (DIN EN ISO 8586) and they have been observed to occur simultaneously in some foods (Chiralertpong et al., 2008; Su et al., 2022). This initial sweet impression undergoes a transformation on days 6–8, evolving into a complex blend of “mushroom”, “woody”, and “earthy” notes. These attributes also form an aroma family in the DLG aroma wheel (DIN EN ISO 8586). Samples obtained after five and nine days of fermentation were characterized by “rancid” and “animalistic” off-flavors. This observation underlines a discernible change in the aroma impression over the course of fermentation.

### 3.4 Combination of analytical and sensory data

A range of analyses was conducted to combine the analytical and sensory data. A partial least squares discriminant analysis (PLS-DA) confirmed that a clear distinction can be made between the two mushrooms based on the aroma compounds detected by GC-MS and the odor attributes selected by the panelists (Q^2^cum = 0.616; R^2^Y = 0.837). The most significant variable importance in the projection (VIP) factors for differentiation in the analytical data are nerolidol (VIP = 1.853), 1-undecanol (VIP = 1.339), and linalool (VIP = 1.157) for AXA as well as carveol (VIP = 1.606), 1-octanol (VIP = 1.492) and 2,4-nonadienal (VIP = 1.424) for LED. These findings are consistent with the fact that some of the aroma compounds mentioned are produced by only one of the two mushrooms. With regard to the sensory analysis, the attributes “sweet” (VIP = 1.302), “caramel” (VIP = 1.158), and “malty” (VIP = 1.071) for AXA and “animalistic” (VIP = 1.308), “musty” (VIP = 1.129), and “grass” (VIP = 1.052) for LED were of particular importance for distinguishing the mushrooms. This finding aligns with the results of the CATA analysis previously outlined.

**Fig. 3:**
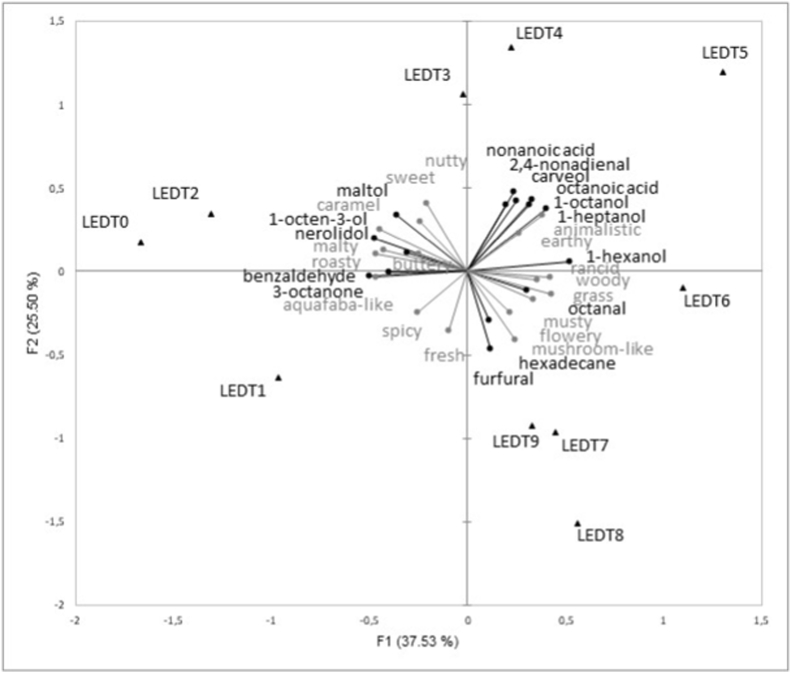
PCA plot of the analytical and sensory results for LED-fermented aquafaba.

To demonstrate correlations between aroma compounds and sensory attributes, a principal component analysis was conducted. Comparable to the correspondence analysis, the principal component analysis did not reveal any discernible trends with regard to the fermentation process with AXA. Furthermore, the first two factors explain only 40.55% of the variability, which suggest that complex relationships arise as a result of the fermentation of aquafaba with AXA. The principal component analysis for LED-fermented aquafaba is shown in Fig. 3. The biplot, which comprises the first two factors, explained 63.02% of the variation. As demonstrated by the correspondence analysis of the CATA data, a separation of the fermentation days by the y-axis can be observed. The fermentation days ranging from the day of inoculation to day 3 are situated to the left of the y-axis, while those from 4 to 9 are positioned to the right. It is also noteworthy that the samples exhibit a tendency to follow a curve around the origin, which suggests that the fermentation process from day 0 to day 9 can be explained by the combination of analytical and sensory data. This difference in behavior between AXA and LED is consistent with the findings reported in 3.3. Whereas certain aroma compounds (e.g., 1-octen-3-ol) and sensory attributes (e.g., aquafaba-like) showed evident trends in LED, this was not observed in AXA. Maltol is known to impart a caramel-like and sweet aroma to food (Buettner, 2017), and in this study a strong positive correlation between maltol and these two attributes was observed (“caramel”: r = 0.78; “sweet”: r = 0.64). In addition, the aroma of maltol is described as reminding of roasted nuts (Rottiers et al., 2019), thereby corroborating the positive correlation with the attributes “roasty” (r = 0.56) and “nutty” (r = 0.51). Furthermore, maltol is an important aroma compound in malt (Su et al., 2022), which may also explain the positive correlation with the attribute “malty” (r = 0.57). As previously stated, a significant (p < 0.0001) decline in maltol content was observed between days 4 and 5. The frequency of selection for the attributes “nutty”, “malty” and “caramel” also decreased between these days. This effect was particularly noticeable for the attribute malty, suggesting that maltol may be primarily responsible for the malty perception. Benzaldehyde, recognized for its almond-like, nutty, and sweet aroma (Li et al., 2019), has also been found to be positively correlated with the aforementioned attributes “roasty” (r = 0.52) and “caramel” (r = 0.49). The aroma compound was detected in high quantities, particularly on fermentation days 1 and 2, which cannot be directly linked to the attributes, but the attribute “sweet” was selected most frequently on day 2. Contrary to expectations, the aroma compound 1-octen-3-ol was found to correlate positively with the “caramel” (r = 0.83), “malty” (r = 0.76), and “roasty” (r = 0.69) odor perceptions. This derivative has been identified as a key contributor to the mushroom flavor (Hofrichter, 2011), although it demonstrated a negative correlation (r = −0.5) with this attribute. This phenomenon can be attributed to the decline in the concentration of 1-octen-3-ol during the fermentation process. The same negative correlation was shown for 3-octanone (r = −0.40), another key compound of mushroom odor that was detected exclusively during the initial two days of fermentation. However, the panelists selected the attribute “mushroom-like” from day 4 to day 8 with increasing frequency, and particularly frequently between day 6 and day 8.

During the sensory training, the panelists trained with individual aroma substances such as 1-octen-3-ol, which should have facilitated familiarity with the specific smell. However, it is crucial to note that the panelists examined the fermented samples in their whole complexity rather than the individual aroma compounds. The combination of aroma compounds in a mixture is known to yield a distinct olfactory experience when compared to the simple addition of individual aroma substances (Buettner, 2017). Furthermore, individuals exhibit different sensitivities to various aroma compounds. Consequently, the predominant olfactory impression of a blend of multiple aroma compounds may vary between individuals (Buettner, 2017). In addition to the attribute “mushroom-like”, the samples from days 6–8 were also described with the attributes “woody” and “earthy”. These terms are closely related to each other, as they can be grouped together in one aroma class (DIN EN ISO 8586). This may have prompted the panelists to also associate the resulting aroma with mushroom flavor, which may have resulted in an increased frequency of selection. Moreover, the perception of individual aroma substances depends on their odor threshold (Buettner, 2017). Depending on the day of fermentation, variations in the aroma substances may be present and perceivable by the olfactory sense.

The attributes “woody” (r = 0.78) and “grass” (r = 0.56) were found to correlate positively with the aroma compound 1-hexanol, which has previously been characterized as grass/green (Zhang et al., 2014). The aroma compound octanal also showed a positive correlation with the attribute woody (r = 0.49). An examination of the CATA data revealed that the attribute “grass” and “woody” were scarcely mentioned at the onset of fermentation, reaching its initial peak after five days and six days respectively. Both aroma compounds, octanal and 1-hexanol, demonstrated a comparable upward trend at the beginning of the fermentation process. On the fifth day of fermentation, 1-hexanol also reached its maximum concentration, while octanal achieved its second highest point. Even though octanal shows only a slight positive correlation with the attribute “grass” (r = 0.22), the aroma compound is described as green/grass in the literature (Yin et al., 2021). It should be noted that octanal showed OAV values above one from day 2 until the end of fermentation (especially on day 5 and 7), which may have led to a higher perception of the “woody” and “green” aroma in the late fermentation stage. The “animalistic” and “rancid” off-flavors that were particularly noticeable on days 5 and 9 of fermentation may have resulted from the interaction of several aroma compounds. The attribute “animalistic” demonstrates a strong positive correlation with the aroma compounds carveol (r = 0.86), 1-octanol (r = 0.76), 1-heptanol (r = 0.90), 2,4-nonadienal (r = 0.69), octanoic acid (r = 0.86), and nonanoic acid (r = 0.80). Notably, 2,4-nonadienal and octanoic acid exhibit high OAV values (OAV>1), indicating a substantial contribution to the perceived aroma. Carveol and octanoic acid have been described in the literature as musty and moldy (Schoenauer and Schieberle, 2016; Wagner et al., 2017), whereas 2,4-nonadienal and nonanoic acid have been described as fatty (Czerny et al., 2008; Zhao et al., 2021), which could lead to a rancid or animalistic perception. The contents of carveol, 2,4-nonadienal, octanoic acid, and nonanoic acid were found to be particularly high on fermentation days 2, 4, and 5, whereas the content of benzaldehyde and maltol decreased over this period of fermentation. This may result in the sweetish smell being unable to mask the other off-flavor olfactory impressions on day 5. Furthermore, an increase in the levels of 1-heptanol and 1-octanol on day 4 was observed, which may serve to mask the initial “animalistic” odor, since they are more commonly associated with fruity and soapy notes (Polster and Schieberle, 2015). It is important to note that the current study only quantified 17 aroma compounds, however, additional volatile compounds might also have contributed to the aroma impression of the samples.

## 4 Conclusion

This study provided insights into the aroma profile of fermented aquafaba. Whereas the use of LED for the fermentation of side streams has frequently been described, this is the first time that the aroma profile of fermentation with AXA has been examined. In this work, the distinct stages of fermentation could be distinguished by considering both analytical and sensory data, which may enable specific applications through a variation in fermentation duration. The present findings provide a valuable basis for further research, which is particularly necessary in relation to the fermentation with AXA, as the enzymatic toolset of this mushroom have not yet been studied in great detail. Gas chromatography olfactometry would be a valuable extension of research, as it enables the examination and description of all substances contributing to the aroma. Because numerous chiral aroma compounds were detected in fermented aquafaba, the determination of the corresponding enantiomeric ratios by enantioselective GC could provide hints to possible formation pathways (enzymatic *versus* non-enzymatic pathways). A sensory evaluation of the aroma liking would also be interesting, as the CATA data suggests that AXA produces a preferred aroma. In conclusion, the present study demonstrates that the fermentation of aquafaba resulted in a decline in the intensity of the characteristic beany odor. This is primarily attributed to the generation of new aroma impressions such as sweet, roasty and woody notes by the basidiomycetes.

## CRediT authorship contribution statement

Lea Mehren: Conceptualization, Methodology, Investigation, Formel analysis, Writing – original draft. Rabea Hondrich: Methodology, Investigation, Formel analysis. Fabian Maretzky: Methodology. Andreas Schieber: Resources, Supervision, Writing – review & editing. Matthias Wüst: Resources, Writing – review & editing. Nadine Schulze-Kaysers: Conceptualization, Supervision, Methodology, Writing – review &editing.

## Ethical statement

No ethics application was submitted for the sensory study, since no tasting took place, as the participants only had to evaluate the samples by smell. Prior to the begin of the study, the panel participants were informed about the study procedure. The participants were informed that they were at liberty to withdraw from the study without the obligation to provide a justification for their decision. The panel participants were provided with a unique code that enabled them to access the questionnaires, thereby ensuring the anonymity of personal data such as age and gender.

## Funding

This research did not receive any specific grant from funding agencies in the public, commercial, or not-for-profit sectors.

## Declaration of competing interest

The authors declare that they have no known competing financial interests or personal relationships that could have appeared to influence the work reported in this paper.

## Data Availability

Data will be made available on request.
